# Relationship between core self-evaluation and innovative work behavior: mediating effect of affective organizational commitment and moderating effect of organizational learning capacity

**DOI:** 10.3389/fpsyg.2023.1192859

**Published:** 2023-10-31

**Authors:** Eunbi Choi, Junhee Kim, Daeyeon Cho

**Affiliations:** ^1^Graduate School of Education, Korea University, Seoul, Republic of Korea; ^2^Department of Education, Korea University, Seoul, Republic of Korea

**Keywords:** core self-evaluation, innovative work behavior, affective organizational commitment, organizational learning capacity, South Korea

## Abstract

Focusing on employees, this study examined the respective mediating and moderating effects of affective organizational commitment and organizational learning capacity in the relationship between core self-evaluation and innovation work behavior. We collected data *via* an online survey from 330 office workers at midsize and large companies in a metropolitan area of South Korea. The results of analyzing the data using PROCESS macro were as follows: (1) core self-evaluation was positively related to innovative work behavior; (2) the relationship was mediated by affective organizational commitment; (3) the relationship was buffered by organizational learning capacity, such that a higher level of organizational learning capacity diminished the impact of core self-evaluation on innovative wok behavior; and (4) the conditional effect of core self-evaluation on innovative work behavior existed only in the group of a low level of organizational learning capacity. Based on these findings, we suggested implications for theory building, research, and practice.

## Introduction

In a rapidly changing business environment, innovation is an important factor for organizational survival and growth (Attiq et al., 2017). In this context, organizational innovation begins with innovative work behavior among employees, who thereby catalyze the process of realizing useful and new ideas for the organization (Tang et al., 2019). For this reason, an increasing number of researchers are focusing on factors that influence employees’ innovative work behavior. Specifically, the influence occurs through interactions between very complex and multi-layered factors within a given organization (Janssen, 2000; Saeed et al., 2019). From this perspective, it is necessary to consider not only the characteristics of individual members (i.e., the source of innovative work behavior), but also the various interactions with organizational factors that can influence individual characteristics and work behaviors (Jafri, 2010; Attiq et al., 2017).

Core self-evaluation is a personal characteristic that can influence innovative work behavior. It is defined as a personal disposition that reflects individuals’ beliefs about their own worth, competence, and control (Judge et al., 1998). As a broad concept, core self-evaluation integrates a variety of personal characteristics, including self-esteem, generalized self-efficacy, locus of control, and emotional stability (Judge et al., 1998; Saeed et al., 2019; Ding and Yu, 2020). For employees, this action reflects individual job behaviors and attitudes, and can increase the potential for innovative work behaviors that require voluntary efforts. Although some empirical studies have shown that core self-evaluation can promote favorable work outcomes such as innovative work behavior (e.g., Attiq et al., 2017; Purba and Paundra, 2018; Zhang et al., 2020), there is a comparative lack of empirical evidence and theories on the underlying mechanism. In order to reveal the complex relationship between core self-evaluation and innovative work behavior, further research on intervening variables (i.e., mediators and moderators) based on sound theories needs be conducted.

Human motivation is an important psychological mechanism that explains the process by which personal characteristics are expressed in behavior (Harter, 1990; Weinstein and Ryan, 2011; Deci et al., 2017). In particular, innovative work behavior requires individuals to engage in additional voluntary efforts that are based on understandings of both the job itself and organizational goals (Marques et al., 2014). Therefore, affective organizational commitment (i.e., an attitude of organizational dedication via voluntary motivation) may work as an intermediating mechanism that more clearly explains the relationship between core self-evaluation and innovative work behavior. In this regard, previous studies revealed the relationships between core self-evaluation and affective organizational commitment (Cadiz, 2010; Joo et al., 2012; Kittinger et al., 2020) and between affective organizational commitment and innovative work behavior (Ng et al., 2010; Nazir et al., 2018; Battistelli et al., 2019; Yuan and Ma, 2022), respectively. However, those empirical evidence cannot verify the mediating role of affective organizational commitment on the relationship between core self-evaluation and innovative work behavior because the three factors have not been analyzed in an identical research model based on a sound theoretical framework.

On the other hand, trait activation theory (Tett and Burnett, 2003; Tett et al., 2021), which explains the mechanism by which personal traits are expressed as behaviors, posits that relationships between personal traits and behaviors can be strengthened or weakened depending on situational conditions. According to this theory, both the individual and environment closely interact and influence individual behavior (Tett et al., 2021). In other words, environmental factors (e.g., organizational learning capacity) within the organization can moderate the intensity of individual characteristics that are manifested by behaviors therein. According to the trait activation theory, organizational learning capacity (i.e., an organizational environment that promotes organizational learning) may act as a moderator on the relationship between core self-evaluation and innovative work behavior. In previous studies (Wang and Ellinger, 2011; Park et al., 2014; Gozukara et al., 2016; Lin and Lee, 2017; Türk and Biçer, 2018; Sari and Palupiningdyah, 2020; Yuan and Ma, 2022), organizational learning capacity was found to contributes to innovative work behavior by promoting the development of creative ideas and various social interactions. Additionally, Aboobaker and Zakkariya (2021) found that organizational learning capacity was a significant moderating variable in the relationship between personal characteristics, including digital learning orientation and readiness for change, and innovative work behavior. The theory and empirical evidence suggest that there is room to deepen the understanding of the moderating role of organizational learning capacity on the relationship between core self-evaluation and innovative work behavior.

A theory development is an ongoing process and further research related to the theory needs to be accumulated to increase its explanatory power, while the theory should guide relevant research to produce robust findings on a specific topic (Lynham, 2002). Focusing on employees in companies in South Korea, this study investigated the respective mediating and moderating effects of affective organizational commitment and organizational learning capacity on the relationship between core self-evaluation and innovative work behavior, by drawing upon relevant theories. The findings may contribute to better theoretical understandings of how core self-evaluation can impact employees’ innovative work behavior, with implications for future research on factors that influence the behavior. Finally, this study highlights the need for organizational-level efforts to build organizational learning capacity from a practitioners’ perspective.

## Theoretical backgrounds and hypothesis development

Job demands-resources theory (Bakker and Demerouti, 2017) and trait activation theory (Tett and Burnett, 2003; Tett et al., 2021) were integrated to establish a theoretical framework of the current study encompassing three hypotheses. First, we drew upon job demands-resources theory to hypothesize the mediating role of affective organizational commitment on the relationship between core self-evaluation and innovative work behavior. To build the theoretical framework, we adopted only the positive motivational process in the theory because our research focus was not on job demands or strain but on a motivational state (i.e., affective organizational commitment) and job performance-related outcome (i.e., innovative work behavior). Second, we used trait activation theory to investigate the moderating role of organizational learning capacity on the relationship between core self-evaluation and innovative work behavior.

### Relationship between core self-evaluation and innovative work behavior

Core self-evaluation is defined as a set of individual dispositional characteristics that indicate an individual’s beliefs about one’s own worth, abilities, competencies, and control over life (Judge et al., 1998, 2003). Thus, core self-evaluation influences the overall perception of the individuals and external environments, implying a strong relationship with organizational behaviors and job-related variables (Chang et al., 2012; Di Fabio et al., 2012; Morris et al., 2013; Attiq et al., 2017; Tang et al., 2019; Yoo and Lee, 2019). Employees with high core self-evaluation positively perceive their own abilities, values, and level of control over life, thus strengthening personal motivation related to positive job attitudes and work behaviors (Saeed et al., 2019).

Meanwhile, innovative work behavior refers to a series of processes and actions in which organizational members create, promote, implement, and apply new ideas in relation to individual performance (Janssen, 2000). For employees, the manifestation of innovative work behavior requires their positive assessment of self-worth and competence that are not only related to their current job, but also their voluntary efforts to develop, promote, and realize creative ideas beyond the current job (Marques et al., 2014). Hence, it is reasonable to predict that employees with a higher level of core self-evaluation are likely to engage in more innovative work behavior as a voluntary effort.

Previous studies have focused on core self-evaluation as a personal characteristic that affects employees’ innovative work employee behavior. For example, the positive relationship between core self-evaluation and innovative work behavior was found in a sample of Pakistani workers (Attiq et al., 2017) and among workers at small enterprises in Jakarta (Purba and Pandura, 2018). These empirical evidence suggests that core self-evaluation is positively related to innovative work behavior among employees. Based on the previous studies (Attiq et al., 2017; Purba and Pandura, 2018), the following hypothesis was established:

*H1*: Employees’ core self-evaluation is positively related to their innovative work behavior.

### Affective organizational commitment as a mediator

Affective organizational commitment refers to an attitude in which an individual has a sense of unity with organizational goals and values and feels a psychological attachment to the organization (Meyer and Allen, 1997). From this perspective, it is the most spontaneous and active form of organizational commitment (Meyer and Allen, 1997). According to job demands-resources theory (Bakker and Demerouti, 2017), employees’ positive perceptions of themselves, self-worth, and competence can act as the employees’ psychological resources that affect their organization-related motivation such as affective organizational commitment. Additionally, the theory suggests that affective organizational commitment as a motivational state mediates the impact of core self-evaluation on innovative work behavior as a job performance-related variable (Bakker and Demerouti, 2017). Consequently, employees with positive core self-evaluation are likely to feel higher affective organizational commitment, which in turn will lead to their active innovative work behavior. These theoretical assumptions are partially supported by several empirical studies as reviewed below.

In previous research, the positive relationship between core self-evaluation and affective organizational commitment was found among nurses affiliated with Oregon Nurses Association in the United States (Cadiz, 2010), workers at South Korean companies (Joo et al., 2012), and MBA students at a university in the United States (Kittinger et al., 2020). Regarding the relationship between affective organizational commitment and innovative work behavior, a positive relationship was found in an Italian military organization (Battistelli et al., 2019), Chinese public sector hospitals (Nazir et al., 2018), national sample of companies (Ng et al., 2010), and Chinese companies (Yuan and Ma, 2022). Although these previous findings may imply a mediating role of affective organizational commitment on the relationship between core self-evaluation and innovative work behavior, the role needs to be investigated by incorporating the three variables in an identical research model. The premise of job demands-resources theory and the previous empirical findings led to the following hypothesis:

*H2*: Employees’ affective organizational commitment mediates the positive relationship between their core self-evaluation and innovative work behavior.

### Organizational learning capacity as a moderator

According to trait activation theory (Tett and Burnett, 2003; Tett et al., 2021), personal trait factors such as temperament and personality manifest as behaviors that are regulated by situations and conditions. Hence, individual characteristics do not consistently influence specific behaviors in all situations, but their exertions become stronger or weaker depending on the situation (Tett et al., 2021). For example, if an expected desirable behavior is supported by sufficient resources and favorable conditions, then individuals will perform this behavior based on their perception of the situation rather than dispositional characteristics. By contrast, individual inclinations have greater influences on behavior under weak conditions where such expectations cannot be met (Beaty et al., 2001). In other words, individual inclinations have limited impacts in situations where a certain factor that promotes a specific behavior is strong, but become more influential when the degree of that factor is relatively weak. In turn, one can reduce the potential for behavioral differences that arise due to individual characteristics by promoting the desired behavior through adequate support and ensuring a high degree of the facilitating factor (Tett et al., 2021).

From the socio-cognitive perspective, organizational learning is defined as a social process in which organizational knowledge is constructed through interactions between members, who constitute a collective body on behalf of the organization (Cho et al., 2013). The process of active social exchange and interaction among members bridges learning at the individual and organizational levels. Here, organizational learning capacity refers to organizational characteristics that promote organizational learning, forming an organizational environment that strengthens social interaction and knowledge socialization (Cho et al., 2013). Under trait activation theory, organizational learning capacity can work as an influential situational factor that promotes active social interactions among organizational members, develops innovative ideas through knowledge socialization, and supports a smooth implementation process thereof. Based on this, core self-evaluation should have a weaker influence on innovative work behavior in organizations with a higher level of organizational learning capacity. In other words, the potential for innovative work behavior increases when organizations have well-established organizational learning capacities, even if employees do not demonstrate their individual traits. By contrast, innovative work behavior requires a higher activation of individual characteristics when organizational learning capacity is not well-equipped; accordingly, core self-evaluation has a greater influence on innovative work behavior. In this context, organizational learning capacity moderates the influence of core self-evaluation on innovative work behavior through a buffering effect, such that a higher level of the capacity reduces the positive influence in that direction.

Previous studies have examined a concept similar to organizational learning capacity as an environmental factor that buffers the influence of individual characteristics. For example, Aboobaker and Zakkariya (2021) found a moderating effect of organizational learning culture that weakened the positive influence of digital learning orientation on innovative behavior. Joo and Shim (2010) revealed that organizational learning culture moderated the relationship between individual self-determination and organizational commitment, such that a higher level of the culture decreased the effect of the former on the latter. In a weak learning culture, individual characteristics will have a stronger influence on organizational commitment. Based on these studies (Joo and Shim, 2010; Aboobaker and Zakkariya, 2021) and trait activation theory (Tett and Burnett, 2003; Tett et al., 2021), we developed the following hypothesis.

*H3:* Organizational learning capacity moderates the relationship between employees’ core self-evaluation and innovative work behavior.

**Figure 1:**
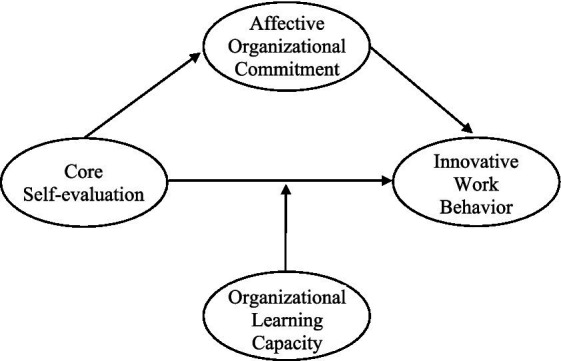
The research model.

Combining the assertions from H1 to H3, the present study hypothesizes that core self-evaluation have a positive impact on innovative work behavior, which is mediated by affective organizational commitment and moderated by organizational learning capacity, respectively. Figure 1 illustrates these hypotheses in a research model.

## Method

### Sample and data collection

The study sample included office workers at midsize and large companies with more than 300 employees in a metropolitan area of South Korea. Office workers perform intellectual activities that realize various duties related to planning, execution, evaluation, information management, and decision-making, all of which aid in the achievement of organizational goals. Today, the high demand for learning has attracted great interest in constant knowledge acquisition and personal development, especially in response to changing environments. This suggests that employees have strong potentials to participate in the process of innovative work behavior within their organizations.

The participants were recruited through the South Korea online research panel Macromill Embrain, which has secured more than 1.6 million panels and conducted professional online research. Availing of panels *via* email, questionnaires were randomly sent to office workers aged 20 years or older at metropolitan-area companies. Data was collected for a period of 1 week, aiming for more than 300 participants to satisfy data normality and achieve the minimum sample size for analysis. A total of 330 valid responses were finally collected.

Looking at the main demographic characteristics of the study sample, 193 participants were male (58.5%) and 137 were female (41.5%). As for age, 104 were in their 50s (31.5%), followed by 83 in their 40s (25.2%), 79 in their 30s (23.9%), and 64 in their 20s (19.4%). Organizational positions included 89 staff (27.0%), 64 department managers (19.4%), 63 general managers (19.1%), 56 assistant managers (17.0%), 49 deputy general managers (14.8%), and 9 executives (2.7%). Regarding length of service in the current workplace, 113 participants reported less than 5 years (34.3%), 99 reported more than 10 years and less than 20 years (19.8%), 54 reported more than 5 years and less than 20 years (16.2%), and 10 reported more than 30 years (13.5%). The participants’ educational levels consisted of 10 high school graduates or below (3%), 33 junior college graduates (10%), 224 college graduates (67.9%), 54 master’s degrees (16.4%), and 9 doctorate degrees (2.7%).

### Measurements

#### Core self-evaluation

Core self-evaluation was measured using 12 items developed by Judge et al. (2003). We used the Korean version of the instrument from Han and Lee (2020) who had translated Judge et al. (2003)’s work into Korean. This instrument consisted of a single factor measured by sample items such as “I am confident I get the success I deserve in life,” “I complete tasks successfully,” “I am filled with doubts about my competence,” and “I do not feel in control of my success in my career.” All items were rated on a 5-point Likert scale ranging from 1 (strongly disagree) to 5 (strongly agree).

#### Affective organizational commitment

Affective organizational commitment was measured using eight items from the multidimensional organizational commitment scale developed by Meyer and Allen (1997). We used the Korean version from Kim et al. (2022) who had translated Meyer and Allen (1997)’s work into Korean. This scale consisted of a single factor measured by sample items such as “I would be very happy to spend the rest of my career in this organization,” “I really feel as if this organization’s problems are my own,” “I do not feel emotionally attached to this organization,” and “I do not feel a strong sense of belonging to my organization.” All items were rated on a 5-point Likert scale ranging from 1 (strongly disagree) to 5 (strongly agree).

#### Organizational learning capacity

Organizational learning capacity was measured using 25 items developed in Korean by Cho et al. (2013). The scale consists of five subfactors, including shared vision and collaborative activity (5 items), personal development (4 items), leadership (6 items), participative decision-making (6 items), and feedback (4 items). Sample items include “My colleagues share the company vision and from a consensus” (shared vision and collaborative activity), “My company encourages employees to continue learning” (personal development), “My boss actively embraces suggestions from employees” (leadership), “My company provides employees with a variety of opportunities to participate in the decision-making process” (participative decision-making), and “My colleagues routinely exchange feedback on each other’s work” (feedback). All items were rated on a 5-point Likert scale ranging from 1 (strongly disagree) to 5 (strongly agree).

#### Innovative work behavior

Innovative work behavior was measured using nine items developed by Janssen (2000). We used the Korean version of Shin and Cho (2017) who had translated Janssen’s (2000) scale into Korean. The scale was used to measure three subfactors, including idea development (3 items), idea promotion (3 items), and idea realization (3 items). Sample items included “I create new ideas for difficult issues” (idea development), “I mobilize support for innovative ideas” (idea promotion), and “I transform innovative ideas into useful applications” (idea realization). All items were rated on a 5-point Likert scale ranging from 1 (strongly disagree) to 5 (strongly agree).

#### Analyses

We used IBM SPSS 26.0 to conduct correlation and reliability analyses, with descriptive statistics for variables. To test the validity of the measurements, the AMOS 23.0 program was used to conduct a confirmatory factor analysis (CFA). Next, the Process macro 4.1 program developed by Hayes (2013) was used to verify the three hypotheses. We chose model 5 in the Process macro program to test the mediating effect and moderated direct effect through bootstrapping (*n* = 10,000) with 95% confidence interval (CI). We controlled any possible effects of the demographic variables (i.e., gender, age, positions, length of service, and educational levels) by adding them as covariates to the analysis model. Finally, to investigate the significance of the conditional effect, the significance area was visualized using the Johnson-Neiman technique through the Process macro 4.1 program.

## Results

### Descriptive statistics, correlations, reliability, and validity

Table 1 presents descriptive statistics, correlation analysis results, internal consistency reliability, and discriminant validity for the variables. The correlation coefficient of each variable showed a weak to moderate correlation, ranging from 0.260 to 0.525. The kurtosis of each variable was less than 0.512 in absolute value, while skewness was less than 0.248 in absolute value, which satisfied the standard of normality. The Cronbach’s alpha values for each variable were 0.806 or higher, indicating internal consistency of items.

**Table 1:** Descriptive statistics, correlations, reliability, and discriminant validity.

	CSE	AOC	OLC	IB
CSE	1			
AOC	0.308^**^	1		
OLC	0.260^**^	0.525^**^	1	
IB	0.363^**^	0.494^**^	0.504^**^	1
Mean	3.240	3.033	3.170	3.227
SD	0.624	0.783	0.629	0.703
Skewness	−0.262	−0.316	−0.327	−0.512
Kurtosis	−0.248	−0.147	0.173	0.166
Cronbach’s alpha	0.856	0.806	0.925	0.927

*n* = 330, *p < 0.01, **p < 0.001; CSE, core self-evaluation; AOC, affective organizational commitment; OLC, organizational learning capacity; IB, innovative work behavior.

**Figure 2:**
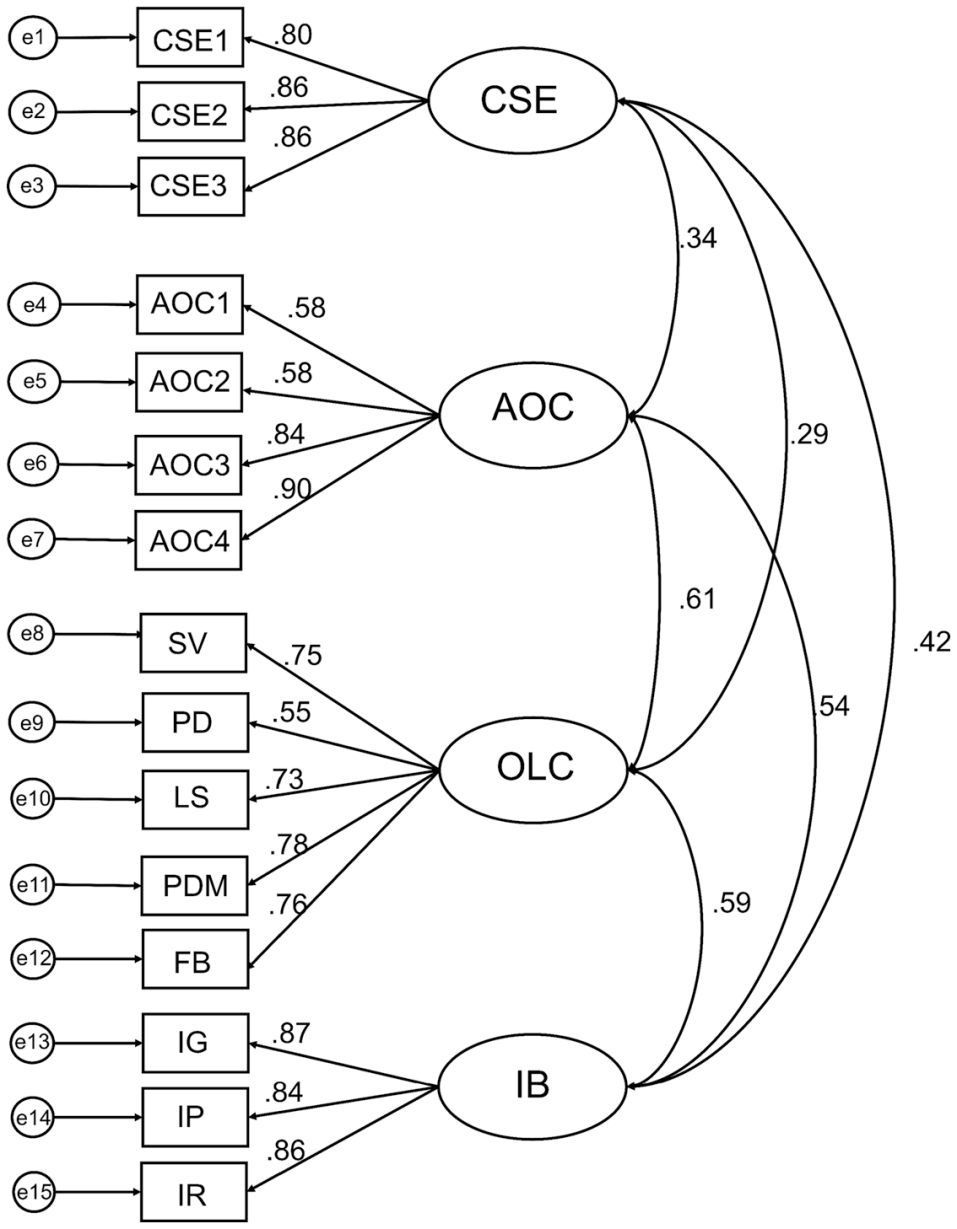
Measurement model analysis results. CSE, core self-evaluation; AOC, affective organizational commitment; OLC, organizational learning capacity; SV, shared vision and collaborative activity; PD, personal development; LS, leadership; PDM, participative decision making; FB, feedback; IB, innovative work behavior; IG, idea generation; IP, idea promotion; IR, idea realization.

Table 2 and Figure 2 present the results of the CFA. Prior to the CFA for the measurement model, the dimensionality of each scale was confirmed through independent CFAs for each measurement scale. Item parceling was also conducted to ensure stable parameter estimation and model fit improvement (Little et al., 2013). As core self-evaluation and affective organizational commitment were composed of a single dimension, the items were grouped by random parceling. As organizational learning capacity and innovative work behavior were composed of subfactors, a content-based approach was used so that items were grouped accordingly (Little et al., 2013). As shown in Table 2, the model fit indices met the criteria for the goodness of fit ( χ ^2^ = 205.317, *df* = 84, *p* = 0.001; CFI = 0.953; TLI = 0.941; SRMR = 0.057; RMSEA = 0.067). The range of the standardized factor loadings for each factor was 0.803 ~ 0.862 for core self-evaluation, 0.580 ~ 0.899 for affective organizational commitment, 0.547 ~ 0.781 for organizational learning capacity, and 0.844 ~ 0.866 for innovative work behavior (see Figure 2).

**Table 2:** Goodness of fit indicators for CFA.

Model fit	χ ^2^	*df*	*P*	CFI	TLI	SRMR	RMSEA
Estimate	205.317	84	0.001	0.953	0.941	0.057	0.067
Threshold	–	–	<0.05	>0.90	>0.90	<0.08	<0.08

### Common method bias test

The study data were simultaneously collected through a self-reported survey. This entails the potential for common method bias (CMB), which can distort the relationships between variables (Chang et al., 2010). This study used an unmeasured latent factor technique by adding the first-order common method factor to the measurement model to determine the impact of the common method factor (Podsakoff et al., 2012; Jordan and Troth, 2020). Comsparing the chi-square value of the measurement model with the CMB model, the difference in Chi-Square value was not significant, indicating that the common method effect was negligible [$\Delta \chi^{2}\left(\Delta df\right)=14.164\left(12\right)\text{,}$ *p* > 0.001]. Based on this, common method factors were not added to the structural model.

### Hypothesis testing

As shown in Table 3, variables were entered into model 5 of Process macro 4.1 to analyze the respective mediating and moderating effects of affective organizational commitment and organizational learning capacity in the relationship between core self-evaluation and innovative work behavior. The results showed that core self-evaluation was positively related to innovative work behavior (*B* = 0.379, *p* < 0.01, 95% CI [0.145, 1.013]), which supported H1. Moreover, a bootstrap with 95% CI verified a mediating effect of affective organizational commitment on the relationship between core self-evaluation and innovative work behavior (indirect effect = 0.048, 95% CI [0.014, 0.092]), which supported H2. Next, the interaction term of core self-evaluation and organizational learning capacity was negatively related to innovative work behavior (*B* = −0.143, *p* < 0.05, 95% CI [−0.277, −0.009]). In other words, as the level of organizational learning capacity increased, a buffer effect weakened the relationship between core self-evaluation and innovative work behavior, supporting H3.

**Table 3:** Results of research model analysis.

Variables	Outcome variables									
	AOC					IB				
	*B*	se	*t*	95% CI		*B*	se	*t*	95% CI	
				LLCI	ULCI				LLCI	ULCI
Constant	0.486	0.471	1.034	−0.440	1.413	−0.779	0.793	−0.982	−2.339	0.782
CSE	0.225	0.061	3.672^***^	0.104	0.345	0.579	0.220	2.626^**^	0.145	1.013
AOC						0.212	0.048	4.428^***^	0.118	0.306
OLC						0.480	0.242	1.990^*^	0.005	0.956
CSE × OLC						−0.143	0.068	−2.096^*^	−0.277	−0.009

Indirect effect				
Mediator	Effect	SE	Boot 95% CI	
			LLCI	ULCI
AOC	0.048	0.020	0.014	0.092

Conditional direct effect						
Moderator			Effect	SE	Boot 95% CI	
					LLCI	ULCI
OLC	M-1SD	2.541	0.216	0.066	0.087	0.345
	Mean	3.171	0.126	0.051	0.025	0.226
	M + 1SD	3.800	0.036	0.068	−0.098	0.169

*n* = 330, *p < 0.05, **p < 0.01, ***p < 0.001; CSE, core self-evaluation; AOC, affective organizational commitment; OLC, organizational learning capacity; IB, innovative work behavior; SE, standard error; LLCI, lower limit confidence level; ULCI, upper limit confidence level.

**Figure 3:**
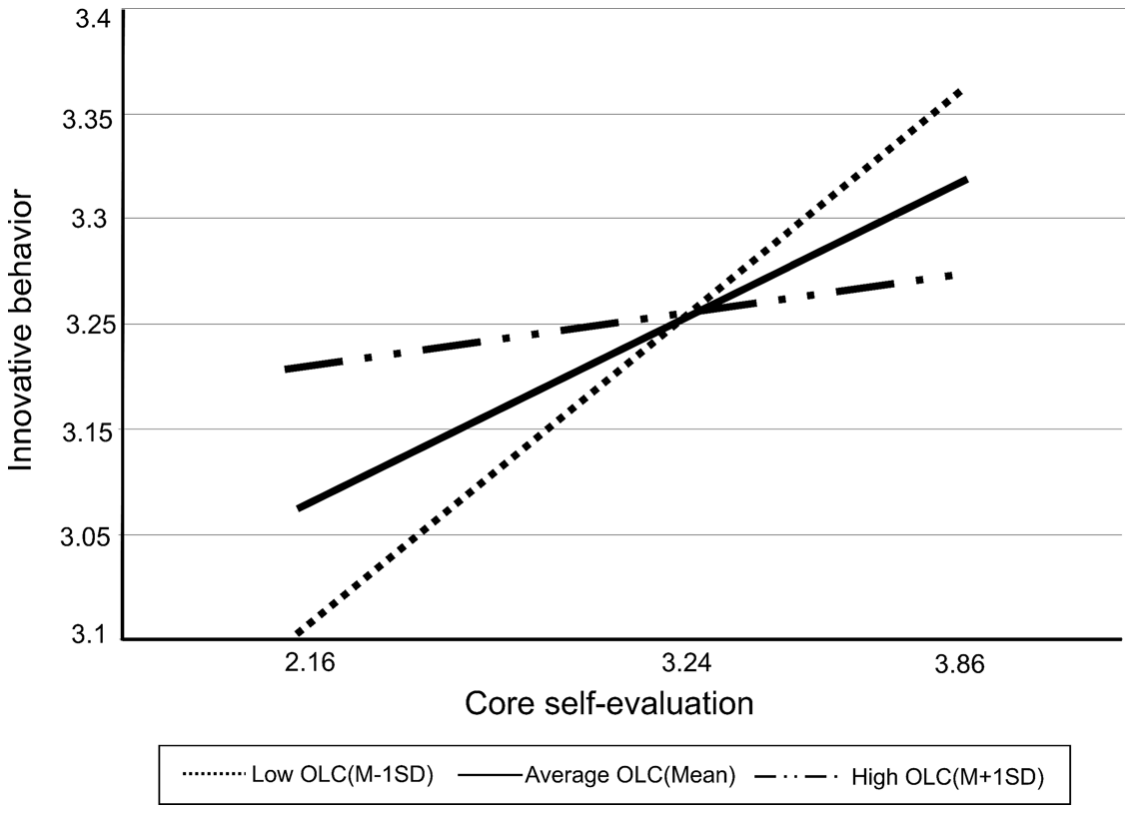
Moderating role of organizational learning capacity.

Finally, the current study analyzed the moderating effect of organizational learning capacity at three levels (M − 1SD, Mean, M + 1SD), as visualized in Figure 3 and shown in Table 3. The result indicated that the relationship between core self-evaluation and innovative work behavior was weakened or strengthened under high or low levels of organizational learning capacity, respectively. The conditional effect of core self-evaluation on innovative work behavior was significant only when organizational learning capacity was at the M-1SD and Mean levels (see Table 3). This indicated that the significance of the conditional effects differed depending on the levels of organizational learning capacity. For this reason, an additional process was needed to search for the specific value of organizational learning capacity that made the conditional effect significant. Therefore, we conducted an interaction probing analysis using the Johnson-Neyman technique to estimate the conditional effect across the entire interval of the continuous moderating variable. The technique enabled us to exclude potential arbitrariness by selecting a specific value (Miller et al., 2013).

**Figure 4:**
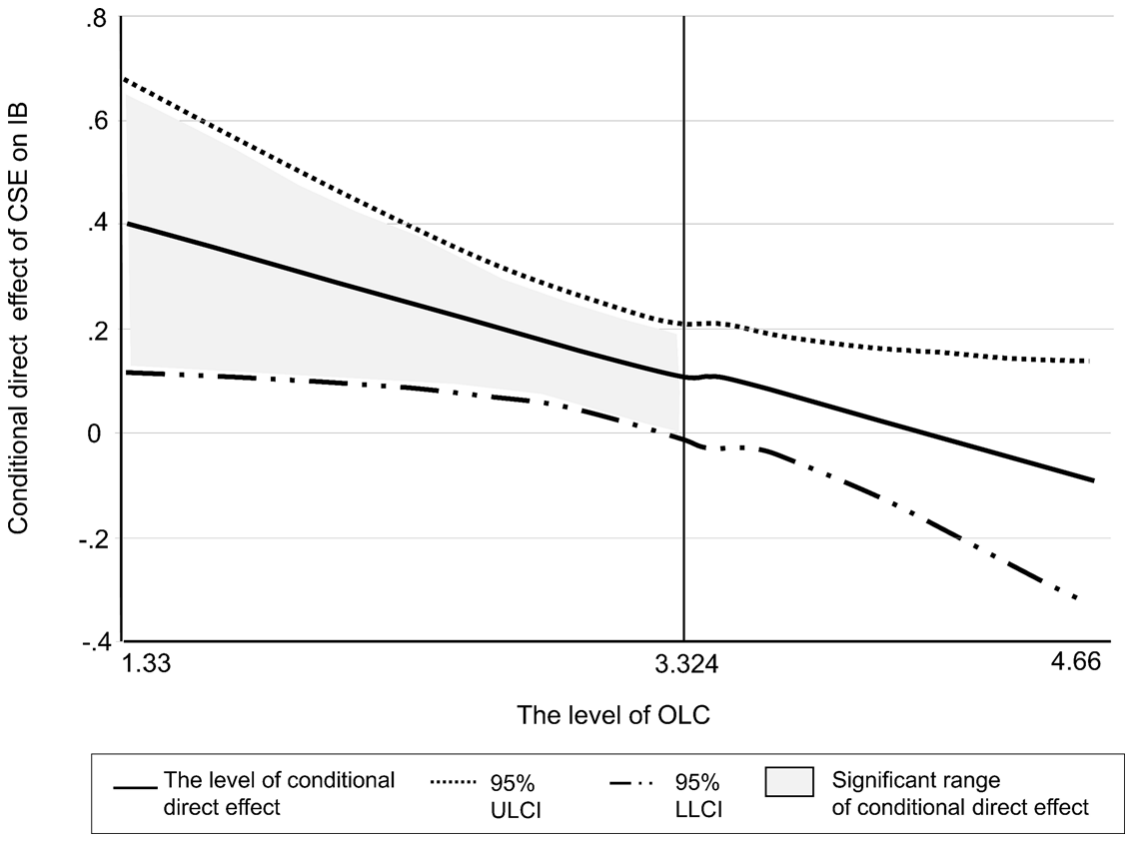
Significant range of conditional direct effect of CSE on IB. CSE, core self-evaluation; OLC, organizational learning capacity; IB, innovative work behavior; LLCI, lower limit confidence level; ULCI, upper limit confidence level.

Table 4 shows the conditional direct effect of core self-evaluation on innovative work behavior depending on the specific value of organizational learning capacity detected *via* the Johnson-Neyman technique. When the value of the mean-centered organizational learning capacity was less than 3.324, the conditional direct effect of core self-evaluation on innovative work behavior was significant, as the 0 was not included in the bootstrap 95% confidence interval. In sum, core self-evaluation significantly impacted innovative work behavior under relatively low levels of organizational learning capacity, while did not have a significant influence under relatively high levels. Figure 4 illustrates the significance of the conditional effect in this direction.

**Table 4:** Conditional direct effect of CSE on IB depending on the levels of OLC.

Moderator	Conditional direct effect	SE	*T*	*p*	Boot 95% CI	
OLC					LLCI	ULCI
**1.333**	**0.388**	**0.134**	**2.905**	**0.004**	**0.125**	**0.651**
**1.500**	**0.364**	**0.123**	**2.957**	**0.003**	**0.122**	**0.607**
**1.666**	**0.341**	**0.113**	**3.014**	**0.228**	**0.118**	**0.563**
**1.833**	**0.317**	**0.103**	**3.076**	**0.002**	**0.114**	**0.519**
**2.000**	**0.293**	**0.093**	**3.140**	**0.002**	**0.109**	**0.477**
**2.166**	**0.269**	**0.840**	**3.203**	**0.002**	**0.104**	**0.434**
**2.333**	**0.245**	**0.075**	**3.257**	**0.001**	**0.097**	**0.394**
**2.500**	**0.222**	**0.067**	**3.285**	**0.001**	**0.089**	**0.354**
**2.666**	**0.198**	**0.061**	**3.260**	**0.001**	**0.078**	**0.317**
**2.833**	**0.174**	**0.055**	**3.140**	**0.002**	**0.065**	**0.283**
**3.000**	**0.150**	**0.052**	**2.880**	**0.004**	**0.048**	**0.253**
**3.166**	**0.126**	**0.051**	**2.466**	**0.014**	**0.026**	**0.227**
3.324	0.104	0.053	1.968	0.050	0.000	0.207
3.333	0.102	0.053	1.940	0.053	−0.002	0.206
3.500	0.079	0.057	1.387	0.167	−0.033	0.190
3.666	0.055	0.062	0.877	0.081	−0.068	0.177
3.833	0.031	0.070	0.445	0.657	−0.106	0.168
4.000	0.007	0.078	0.091	0.928	−0.146	0.160
4.166	−0.017	0.087	−0.194	0.847	−0.187	0.154
4.333	−0.041	0.096	−0.423	0.673	−0.229	0.148
4.500	−0.064	0.106	−0.609	0.543	−0.272	0.144
4.666	−0.088	0.116	−0.762	0.447	−0.316	0.140

Significant range of conditional direct effect of CSE on IB is shown in bold; CSE, core self-evaluation; OLC, organizational learning capacity; IB, innovative work behavior; SE, standard error; LLCI, lower limit confidence level; ULCI, upper limit confidence level.

## Discussion and conclusion

The mechanism by which employees’ core self-evaluation affects their innovative work behavior should reflect a complex relationship of associated variables by integrating both mediating and moderating effects simultaneously. Drawing upon job demands-resources theory and trait activation theory, the current study revealed the respective mediating and moderating effects of affective organizational commitment and organizational learning capacity in the relationship between core self-evaluation and innovative work behavior. This is a significant contribution to the existing body of literature because previous studies have not investigated simultaneously the mediating and moderating roles of the variables in an identical research model, especially based on sound theories. The findings of the current study are discussed in more detail below.

First, core self-evaluation was positively related to innovative work behavior. In other words, a higher level of core self-evaluation (a personal characteristic) resulted in a higher potential for innovative work behavior. This result supports previous studies (Attiq et al., 2017; Purba and Pandura, 2018) that verified the existence of a positive relationship between core self-evaluation and innovative work behavior. While an earlier study by Kim and Koh (2011) reported that core self-evaluation did not have a significant direct effect on innovative work behavior, their investigation targeted a specific occupation of clinical nurses working at a hospital. In this regard, it can be inferred that unique job characteristics and/or distinct organizational factors likely have greater effects on innovative work behavior than individual characteristics.

Second, affective organizational commitment mediated the relationship between core self-evaluation and innovative work behavior. In this arrangement, core self-evaluation had a positive effect on affective organizational commitment, which then had a positive effect on innovative work behavior. These results support Joo et al. (2012) and Kittinger et al. (2020), who reported that core self-evaluation had a positive relationship with affective organizational commitment. They also support Battistelli et al. (2019) and Yuan and Ma (2022), who reported that affective organizational commitment has a positive relationship with innovative work behavior. Based on previous studies (Joo et al., 2012; Battistelli et al., 2019; Kittinger et al., 2020; Yuan and Ma, 2022), these findings demonstrate a new path in which core self-evaluation affects innovative work behavior *via* affective organizational commitment as a mediator.

Third, organizational learning capacity moderated the relationship between core self-evaluation and innovative work behavior. More specifically, organizational learning capacity had a buffering effect that reduced the positive impact of core self-evaluation on innovative work behavior. In other words, core self-evaluation had a lower influence on innovative work behavior under a higher level of organizational learning capacity. As trait activation theory implies, the effects of situational factors related to innovative work behavior become stronger in organizations with a high-level organizational learning capacity; accordingly, individual employee characteristics such as core self-evaluation become less important. This result supports both Aboobaker and Zakkariya (2021) and Joo and Shim (2010), who reported the existence of a moderating effect in which organizational learning impacted the influence of personal dispositions on job behaviors by acting as a strong situational factor. Moreover, this study verified the significant range of the conditional effect of core self-evaluation on innovative work behavior, as moderated by organizational learning capacity. This showed that core self-evaluation only affected innovative work behavior when organizational learning capacity was at a relatively low level. In other words, core self-evaluation positively affects innovative work behavior under relatively low levels of organizational learning capacity, but not under high levels. From the perspective of trait activation theory, this means that organizational learning capacity works as a situational factor that weakens the influence of core self-evaluation on innovative work behavior.

### Implications for theory building and research

A theory building should be an ongoing process based on empirical findings to improve its’ explanatory power for a certain phenomenon because a theory is never complete (Lynham, 2002). The current study was guided by a theoretical framework integrating two theories: job demands-resources theory and trait activation theory. However, we relied solely on empirical findings (e.g., Attiq et al., 2017; Purba and Pandura, 2018) to hypothesize the direct relationship between core self-evaluation and innovative work behavior (H1) because the two theories did not depict the relationship. Thus, we recommend that the theories incorporate our findings in their propositions. For example, a direct relationship between personal resources (i.e., core self-evaluation) and job performance (i.e., innovative work behavior) could be proposed in job demands-resources theory. In contrast, researchers will need to investigate a direct relationship between organizational learning capacity (i.e., job resources) and affective organizational commitment (i.e., motivation) as job demands-resources theory suggests (Bakker and Demerouti, 2017).

Additionally, although organizational learning capacity can be regarded as a job resource (Schaufeli and Taris, 2014), we drew upon trait activation theory to examine its’ moderating effect in our research model because job demands-resources theory did not illuminate the moderating effect of job resources in the relationship between personal resources and job performance (Bakker and Demerouti, 2017). Proposition 4 of job demands-resources theory highlights a reciprocal relationship between personal resources and job resources, but this needs to be refined and expanded in order to clarify the interaction between the two categories of variables (Bakker et al., 2023). Consequently, given that organizational learning capacity was found to moderate the relationship between a personal resource (i.e., core self-evaluation) and job performance (i.e., innovative work behavior), this finding needs to be incorporated in the latest version of job demands-resources theory (Bakker et al., 2023).

### Implications for practice

The findings suggest three implications for practitioners. First, organizations should give their employees opportunities for personal development, including job rotation and mentoring. Such provisions can strengthen existing capabilities and support career development paths that enhance positive core self-evaluation. Organizations that respond sensitively to rapid situational changes and require constant innovation should also make efforts to strengthen individual characteristics that promote innovative work behavior. It is because if the level of core self-evaluation increases, then the potential for innovative work behavior increases.

Second, organizations should actively share their goals and values, which can be realized through a supportive system and fair culture that satisfy employees’ expectations. Such an approach will increase employees’ affective organizational commitment that activates their voluntarily devotion. In this context, it is especially important to promote intrinsic motivation, instead of focusing on the cost of turnover or emphasizing a sense of organizational duty. Therefore, organizations that emphasize innovation should also provide specific goals and visions so that members feel a sense of organizational unity and identification. At the same time, they should provide psychological compensations that meet individuals’ needs and expectations.

Third, to promote innovative work behavior, the level of organizational learning capacity should be diagnosed at the outset. Then, various personal and organizational development strategies should be implemented according to the levels diagnosed. It is because organizational learning capacity is a socio-cognitive factor that is difficult to build over a short period of time (Cho et al., 2013). For example, core self-evaluation can strongly influence innovative work behavior in organizations with a low level of organizational learning capacity. This emphasizes the need for providing career development opportunities to enhance a positive core self-evaluation. By contrast, employees in organizations with a high level of organizational learning capacity are less likely to be influenced by their characteristics. In that case, this suggests that organizations increase employees’ innovative work behavior by systematizing the process, system, and culture for organizational learning.

### Limitations

The present study is not free from some limitations. First, the sample consisted of office workers in midsize and large corporations with more than 300 employees in a metropolitan area of South Korea, where innovative work behavior was expected to occur actively. However, small and midsize enterprises with less than 300 employees account for 84% of all domestic companies in South Korea, which makes it difficult to generalize the current results to all companies in the nation. Second, a correlation was observed between affective organizational commitment and organizational learning capacity, but we did not consider the relationship because of our theoretical framework. Finally, the current study did not distinguish the levels of variables in the research model. Because organizational learning capacity is an organization-level variable, future researchers are recommended to apply a multi-level approach to their research design when investigating relationships among the variables.

## Data availability statement

The original contributions presented in the study are included in the article/supplementary material, further inquiries can be directed to the corresponding author.

## Author contributions

EC, JK, and DC: conceptualization and validation. EC: methodology, formal analysis, writing—original draft preparation, and project administration. JK and DC: writing—review and editing. DC: supervision. All authors contributed to the article and approved the submitted version.

## Funding

This research was supported by the College of Education, Korea University Grant in 2023.

## References

[ref1] Aboobaker N. Zakkariya K. A. (2021). Digital learning orientation and innovative behavior in the higher education sector: effects of organizational learning culture and readiness for change. Int. J. Educ. Manag. 35, 1030–1047. doi: 10.1108/IJEM-09-2019-0345

[ref2] Attiq S. Wahid S. Javaid N. Kanwal M. (2017). The impact of employees’ core self-evaluation personality trait, management support, co-worker support on job satisfaction, and innovative work behaviour. Pak. J. Psychol. Res. 32, 247–271. doi: 10.3389/fpsyg.2022.946362

[ref3] Bakker A. B. Demerouti E. (2017). Job demands–resources theory: taking stock and looking forward. J. Occup. Health Psychol. 22, 273–285. doi: 10.1037/ocp0000056 27732008

[ref4] Bakker A. B. Demerouti E. Sanz-Vergel A. (2023). Job demands–resources theory: ten years later. Annu. Rev. Organ. Psych. Organ. Behav. 10, 25–53. doi: 10.1146/annurev-orgpsych-120920-053933

[ref5] Battistelli A. Odoardi C. Vandenberghe C. Di Napoli G. Piccione L. (2019). Information sharing and innovative work behavior: the role of work-based learning, challenging tasks, and organizational commitment. Hum. Resour. Dev. Q. 30, 361–381. doi: 10.1002/hrdq.21344

[ref6] Beaty J. C. Jr. Cleveland J. N. Murphy K. R. (2001). The relation between personality and contextual performance in “strong” versus “weak” situations. Hum. Perform. 14, 125–148. doi: 10.1207/s15327043hup1402_01

[ref7] Cadiz D. M. (2010). The effects of ageism climates and core self-evaluations on nurses' turnover intentions, organizational commitment, and work engagement. Unpublished doctoral dissertation. Portland, OR Portland State University

[ref9006] Chang S. J. Van Witteloostuijn A. Eden L. (2010). From the editors: Common method variance in international business research. Journal of International Business Studies, 41, 178–184. doi: 10.1057/jibs.2009.88

[ref8] Chang C. H. Ferris D. L. Johnson R. E. Rosen C. C. Tan J. A. (2012). Core self-evaluations: a review and evaluation of the literature. J. Manag. 38, 81–128. doi: 10.1177/0149206311419661

[ref9] Cho D. Y. Eum W. J. Lee K. H. (2013). The impact of organizational learning capacity from the socio-cognitive perspective on organizational commitment. Asia Pac. Educ. Rev. 14, 511–522. doi: 10.1007/s12564-013-9282-9

[ref11] Deci E. L. Olafsen A. H. Ryan R. M. (2017). Self-determination theory in work organizations: the state of a science. Annu. Rev. Organ. Psych. Organ. Behav. 4, 19–43. doi: 10.1146/annurev-orgpsych-032516-113108

[ref12] Di Fabio A. Palazzeschi L. Bar-On R. (2012). The role of personality traits, core self-evaluation, and emotional intelligence in career decision-making difficulties. J. Employ. Couns. 49, 118–129. doi: 10.1002/j.2161-1920.2012.00012.x

[ref13] Ding H. Yu E. (2020). Follower strengths-based leadership and follower innovative behavior: the roles of core self-evaluations and psychological well-being. Rev. Psicol. Trabajo Organ. 36, 103–110. doi: 10.5093/jwop2020a8

[ref15] Gozukara I. Yildirim O. Yildiz B. (2016). Innovative behavior: relations with developmental culture, psychological empowerment, distributive justice and organizational learning capacity. Int. Bus. Res. 9, 186–200. doi: 10.5539/ibr.v9n10p186

[ref16] Han D. S. Lee H. R. (2020). The effects of hotel supervisor’s empowering leadership on job engagement: the dual mediating effect of core self-evaluations and affective commitment. J. Tour. Leisure Res. 32, 271–291. doi: 10.31336/JTLR.2020.3.32.3.271

[ref17] Harter S. (1990). Developmental differences in the nature of self-representations: implications for the understanding, assessment, and treatment of maladaptive behavior. Cogn. Ther. Res. 14, 113–142. doi: 10.1007/bf01176205

[ref18] Hayes AF (2013). Introduction to mediation, moderation, and conditional process analysis. New York, NY: Guilford Press.

[ref19] Jafri M. H. (2010). Organizational commitment and employee's innovative behavior. J. Manag. Res. 10, 62–68.

[ref20] Janssen O. (2000). Job demands, perceptions of effort-reward fairness and innovative work behaviour. J. Occup. Organ. Psychol. 73, 287–302. doi: 10.1348/096317900167038

[ref9007] Jordan P. J. Troth A. C. (2020). Common method bias in applied settings: The dilemma of researching in organizations. Australian Journal of Management. 45, 3–14.

[ref21] Joo B. K. Shim J. H. (2010). Psychological empowerment and organizational commitment: the moderating effect of organizational learning culture. Hum. Resour. Dev. Int. 13, 425–441. doi: 10.1080/13678868.2010.501963

[ref22] Joo B. K. Yoon H. J. Jeung C. W. (2012). The effects of core self-evaluations and transformational leadership on organizational commitment. Leader. Organ. Develop. J. 33, 564–582. doi: 10.1108/01437731211253028

[ref23] Judge T. A. Erez A. Bono J. E. Thoresen C. J. (2003). The core self-evaluations scale: development of a measure. Pers. Psychol. 56, 303–331. doi: 10.1111/j.1744-6570.2003.tb00152.x

[ref24] Judge T. A. Locke E. A. Durham C. C. Kluger A. N. (1998). Dispositional effects on job and life satisfaction: the role of core evaluations. J. Appl. Psychol. 83, 17–34. doi: 10.1037/0021-9010.83.1.17, PMID: 9494439

[ref26] Kim J. W. Choi E. B. Cho D. Y. (2022). The mediation effect of coaching behavior in the relationship between creativity and affective commitment of Army executives. Glob. Creat. Lead. Educat. Learn. 12, 51–82. doi: 10.34226/gcl.2022.12.2.51

[ref27] Kim M. J. Koh M. S. (2011). Mediating effects of self-leadership on the relationship between nurses’ core self-evaluation and innovative behaviour. Health Soc. Sci. 30, 171–198.

[ref28] Kittinger J. D. Walker A. G. Cope J. G. Wuensch K. L. (2020). The relationship between core self-evaluations and affective commitment. J. Behav. Appl. Manag. 11, 68–92. doi: 10.21818/001c.17322

[ref29] Lin H. C. Lee Y. D. (2017). A study of the influence of organizational learning on employees’ innovative behavior and work engagement by a cross-level examination. Eur. J. Math. Sci. Technol. Educat. 13, 3463–3478. doi: 10.12973/eurasia.2017.00738a

[ref30] Little T. D. Rhemtulla M. Gibson K. Schoemann A. M. (2013). Why the items versus parcels controversy needn’t be one. Psychol. Methods 18, 285–300. doi: 10.1037/a0033266, PMID: 23834418 PMC3909043

[ref31] Lynham S. A. (2002). The general method of theory-building research in applied disciplines. Adv. Dev. Hum. Resour. 4, 221–241. doi: 10.1177/1523422302043002

[ref32] Marques T. Galende J. Cruz P. Ferreira M. P. (2014). Surviving downsizing and innovative behaviors: a matter of organizational commitment. Int. J. Manpow. 35, 930–955. doi: 10.1108/ijm-03-2012-0049

[ref33] Meyer J. P. Allen N. J. (1997). Commitment in the workplace: theory, research, and application. Thousand Oaks Sage Publications

[ref35] Miller J. W. Stromeyer W. R. Schwieterman M. A. (2013). Extensions of the Johnson-Neyman technique to linear models with curvilinear effects: derivations and analytical tools. Multivar. Behav. Res. 48, 267–300. doi: 10.1080/00273171.2013.763567, PMID: 26741727

[ref36] Morris M. L. Messal C. B. Meriac J. P. (2013). Core self-evaluation and goal orientation: understanding work stress. Hum. Resour. Dev. Q. 24, 35–62. doi: 10.1002/hrdq.21151

[ref37] Nazir S. Qun W. Hui L. Shafi A. (2018). Influence of social exchange relationships on affective commitment and innovative behavior: role of perceived organizational support. Sustainability 10, 1–20. doi: 10.3390/su10124418

[ref38] Ng T. W. Feldman D. C. Lam S. S. (2010). Psychological contract breaches, organizational commitment, and innovation-related behaviors: a latent growth modeling approach. J. Appl. Psychol. 95, 744–751. doi: 10.1037/a0018804, PMID: 20604593

[ref39] Park Y. K. Song J. H. Yoon S. W. Kim J. (2014). Learning organization and innovative behavior: the mediating effect of work engagement. Eur. J. Train. Develop. 38, 75–94. doi: 10.1108/ejtd-04-2013-0040

[ref9008] Podsakoff P. M. MacKenzie S. B. Podsakoff N. B. (2012). Sources of method bias in social science research and recommendations on how to control it. Annual Review of Psychology, 63, 539–569. 10.1146/annurev-psych-120710-100452 21838546

[ref40] Purba D. E. Paundra J. (2018). Core self-evaluations and innovative behavior among micro-entrepreneurs: the mediating effect of proactive personality. Psychol. Res. Urban Soc. 1, 55–64. doi: 10.7454/proust.v1i1.30

[ref41] Saeed B. B. Afsar B. Cheema S. Javed F. (2019). Leader-member exchange and innovative work behavior. Eur. J. Innov. Manag. 22, 105–124. doi: 10.1108/ejim-11-2017-0158

[ref42] Sari F. K. Palupiningdyah P. (2020). The effect of mediation work engagement to procedural justice and organizational learning on the innovative behavior. Manag. Anal. J. 9, 152–160. doi: 10.15294/maj.v9i2.37011

[ref43] Schaufeli W. B. Taris T. W. (2014). “A critical review of the job demands-resources model: implications for improving work and health” in Bridging occupational, organizational and public health: a transdisciplinary approach. eds. Bauer G. E. Hämmig O. (Berlin: Springer), 43–68.

[ref44] Shin D. J. Cho D. Y. (2017). The moderating effect of critically reflective work behavior on relationship between learning orientation and innovative behavior in a company. J. Korean HRD Res. 12, 165–190. doi: 10.21329/khrd.2017.12.2.165

[ref45] Tang Y. Shao Y. F. Chen Y. J. (2019). Assessing the mediation mechanism of job satisfaction and organizational commitment on innovative behavior: the perspective of psychological capital. Front. Psychol. 10:2699. doi: 10.3389/fpsyg.2019.02699, PMID: 31920781 PMC6928100

[ref46] Tett R. P. Burnett D. D. (2003). A personality trait-based interactionist model of job performance. J. Appl. Psychol. 88, 500–517. doi: 10.1037/0021-9010.88.3.500, PMID: 12814298

[ref47] Tett R. P. Toich M. J. Ozkum S. B. (2021). Trait activation theory: a review of the literature and applications to five lines of personality dynamics research. Annu. Rev. Organ. Psych. Organ. Behav. 8, 199–233. doi: 10.1146/annurev-orgpsych-012420-062228

[ref48] Türk M. Biçer M. (2018). A research on the relationship between ethical climate, organizational learning and innovative behavior. Int. J. Bus. Manag. Econ. Res. 9, 1207–1218.

[ref49] Wang Y. L. Ellinger A. D. (2011). Organizational learning: perception of external environment and innovation performance. Int. J. Manpow. 32, 512–536. doi: 10.1108/01437721111158189

[ref50] Weinstein N. Ryan R. M. (2011). A self-determination theory approach to understanding stress incursion and responses. Stress. Health 27, 4–17. doi: 10.1002/smi.1368

[ref51] Yoo K. Lee K. H. (2019). Core self-evaluation and work engagement: moderated mediation model of career adaptability and job insecurity. Front. Psychol. 10:2093. doi: 10.3389/fpsyg.2019.02093 31620047 PMC6759723

[ref52] Yuan H. Ma D. (2022). Gender differences in the relationship between interpersonal trust and innovative behavior: the mediating effects of affective organizational commitment and knowledge-sharing. Behav. Sci. 12, 1–15. doi: 10.3390/bs12050145 PMC9137488 35621442

[ref53] Zhang Y. Sun J. M. Lin C. H. Ren H. (2020). Linking core self-evaluation to creativity: the roles of knowledge sharing and work meaningfulness. J. Bus. Psychol. 35, 257–270. doi: 10.1007/s10869-018-9609-y

